# Social accountability in primary health care in West and Central Africa: exploring the role of health facility committees

**DOI:** 10.1186/s12913-017-2344-7

**Published:** 2017-06-13

**Authors:** Elsbet Lodenstein, Eric Mafuta, Adolphe C. Kpatchavi, Jean Servais, Marjolein Dieleman, Jacqueline E. W. Broerse, Alpha Amadou Bano Barry, Thérèse M. N. Mambu, Jurrien Toonen

**Affiliations:** 1 Athena Institute for Research on Innovation and Communication in Health and Life Sciences, VU University and KIT Gender, De Boelelaan 1085, 1081 HV Amsterdam, the Netherlands; 2 Kinshasa School of Public Health, Faculty of Medicine, University of Kinshasa, Po Box: 11850, Kinshasa, DR Congo; 3 Sociologue - Anthropologue, Université d’Abomey Calavi, Cotonou, Benin; 4 UNICEF Western and Central Africa Regional Office, PO Box 29720, Dakar, Senegal; 5 KIT Health, PO Box 95001, 1090 HA Amsterdam, the Netherlands; 6 Athena Institute for Research on Innovation and Communication in Health and Life Sciences Communication, VU University, De Boelelaan 1085, 1081 HV Amsterdam, The Netherlands; 7 Observatoire de Sociologie, Université de Sonfonia, PO Box 3334, Conakry, Guinea

**Keywords:** Health facility committee, Community participation, Accountability, Social accountability, Primary health care

## Abstract

**Background:**

Social accountability has been emphasised as an important strategy to increase the quality, equity, and responsiveness of health services. In many countries, health facility committees (HFCs) provide the accountability interface between health providers and citizens or users of health services. This article explores the social accountability practices facilitated by HFCs in Benin, Guinea and the Democratic Republic of Congo.

**Methods:**

The paper is based on a cross-case comparison of 11 HFCs across the three countries. The HFCs were purposefully selected based on the (past) presence of community participation support programs. The cases were derived from qualitative research involving document analysis as well as interviews and focus group discussions with health workers, citizens, committee members, and local authorities.

**Results:**

Most HFCs facilitate social accountability by engaging with health providers in person or through meetings to discuss service failures, leading to changes in the quality of services, such as improved health worker presence, the availability of night shifts, the display of drug prices and replacement of poorly functioning health workers. Social accountability practices are however often individualised and not systematic, and their success depends on HFC leadership and synergy with other community structures. The absence of remuneration for HFC members does not seem to affect HFC engagement in social accountability.

**Conclusions:**

Most HFCs in this study offer a social accountability forum, but the informal and non-systematic character and limited community consultation leave opportunities for the exclusion of voices of marginalised groups. More inclusive, coherent and authoritative social accountability practices can be developed by making explicit the mandate of HFC in the planning, monitoring, and supervision of health services; providing instruments for organising local accountability processes; strengthening opportunities for community input and feedback; and strengthening links to formal administrative accountability mechanisms in the health system.

## Background

Since the 1978 Alma-Ata Declaration on Primary Health Care, participation has been a central theme of health policy and programming. The 2008 World Health Report re-emphasizes the values of community participation to achieve “people-centred” health systems [1]. Health service users are increasingly seen as citizens who should be allowed to voice their concerns actively, to shape health services and policies in the public interest and hold health providers and policymakers accountable [2, 3]. The expectations of this form of accountability referred to as *social accountability* are high, in particular in countries where health systems face persistent service delivery failures [4]. In many low-and-middle-income countries, health facility committees (HFC) are one of the well-known vehicles for community participation. They are involved in the co-management of primary public health facilities, in the spirit of the Bamako Initiative that was launched in 1987 (see Table 1). While the Alma-Ata Declaration expressed the key principles of Primary Health Care, the Bamako Initiative operationalized the principle of community participation in the organisation of health services. It introduced measures that aimed to give users, through representatives in HFCs, say in determining access to services and the use of funds obtained through the sale of drugs. Some HFCs, especially in health centres with high utilisation rates, developed into structures with significant influence on the management of human, financial and material resources needed to provide quality of care [5–7].

**Table 1:** The Bamako initiative

Background	The Bamako Initiative (BI) is a policy statement, adopted in 1987 by African health ministers in Bamako, Mali. It was developed in the context of economic crises and negative effects of adjustment programmes in many Sub-Saharan countries. Formulated by UNICEF and WHO, the initiative aimed to promote universal access to primary health care.
Objectives	• Strengthen the management and financing of health care at the local level. • Promote community participation. • Improve the supply, management and use of essential drugs. • Ensure sustainable financing of primary health care units.
Principles	Decentralisation of decision-making to health districts and of financial management to communities; partial cost-recovery through the sale of essential drugs; sufficient funding for primary healthcare by governments; exemption policies for the poorest groups in society; health promotion and a multi-sectorial approach to health care.

*Source*: Ridde [7]

In many countries, HFCs are attached to health centres and elected by community members to facilitate communication and feedback processes between health providers and citizens or users of health services. They, therefore, have the potential to ensure financial and social accountability of health providers to communities and to strengthen the democratic governance of health systems more generally [8].

Recent literature reviews on HFCs found that the extent to which HFCs in developing countries can influence service provision is mixed. This is due to the diversity in the composition of HFCs, their roles and responsibilities, the availability of resources, as well as the differences in health systems and policies, community and societal contexts in which HFCs operate [4, 9, 10]. These reviews led to a better conceptualization of contextual elements that influence HFC effectiveness, but they also called for more empirical tests of the frameworks. Based on a synthesis of findings from case studies in West and Central Africa, this paper explores the functioning of HFCs, in particular with regard to their actual and potential role in the facilitation of social accountability. A recent systematic review of the literature on community participation by George et al. revealed that most studies on the topic focus on participation in health promotion interventions and effects on service uptake and less on community involvement and empowerment in the governance of health services [11]. This paper aims to address this research gap and to provide recommendations as to how the role of HFCs in social accountability can be enhanced.

### The social accountability role of HFC

Social accountability is a contested concept used in a variety of disciplines, including in the context of professional health education [12], New Public Management [13] and participatory democracy [14]. In the health sector, social accountability is often viewed as an advanced form of community participation whereby citizens take action to enhance the accountability of politicians, policymakers and service providers. The role of HFCs in social accountability has rarely been assessed. HFCs are defined as “any formally constituted structures with community representation that has an explicit link to a health facility and whose primary purpose is to enable community participation with the aims of improving health service provision and health outcomes” [9]. HFCs can exist at several levels and take different forms from village level health committees to community health groups and hospital boards for district hospitals [4]. This study focuses on HFCs at the level of primary healthcare centres offering basic packages of healthcare.

HFCs can perform two sets of activities to improve health service provision, presented in Table 2 as two roles. The first role is to support the functioning of health facilities and the objectives of health providers. HFCs serve as an extension of service providers and engage in community outreach, the co-management of health centre resources and the facilitation of repairs and fundraising. This role is quite prevalent in practices of HFCs in low-and-middle-income countries [10]. McCoy et al. refer to the activities under this role as facing “inwardly” [9]. The second role supports users’ and citizens’ voice and the “bottom-up” integration of community preferences in decision-making in service delivery. McCoy et al. call this the “outward” role [9]. HFCs activities include advocating for access to health care (“social leveller”) or resources (“advocacy”), the monitoring of the quality of care and the use of funds (“control of quality and management”) and the facilitation of feedback mechanisms between health providers and users (“provide accountability interface”). Whether or not, and how HFCs perform these roles varies, as HFCs are complex entities embedded in country-specific political, historical and health system contexts [9, 10]. Moreover, in many countries, HFC members work as unpaid volunteers; HFC effectiveness, then, depends on the personal commitment of individual members.

**Table 2:** Main roles and activities of health facility committees

Main role	Activities
1. Inward role: support health facility & workers	*Co-management* – of health facility resources and services
	*Resource generator –* in the form of material resources, labour and funds for health facility
	*Community outreach* – to help the health facility reach into the community for the purpose of health promotion and improving health-seeking behaviour; organisation of community-based health activities
2. Outward role: facilitating citizen voice and accountability	*Advocacy* – to act as a community voice to advocate (e.g. to local politicians and health managers higher up the health system) on behalf of the health facility
	*Social leveller* - to help mitigate social stratification by defending rights of marginalised sections of the community/public
	*Control of quality and management* – including the monitoring of use and quality of material, financial resources and the performance of health workers, results of health services
	*Provide accountability interface* by initiating and facilitating feedback process between users/citizens and health providers and authorities by following the steps in an accountability cycle: 1. *Information/data collection* – information on performance through monitoring, the collection, interpretation and articulation of users’ and citizens’ views, demands and complaints. 2. *Dialogue/forum* - provide a means to transmit, question and discuss this information to/with health providers and authorities and claim improvements. 3. *Consequences* - follow up on responses and decisions taken and for results; when responses or explanations fail, there should be a possibility to reward/sanction health providers’ actions and results. 4. *Counter feedback* to users’ and citizens.

Source: adapted from [9, 15]

**Fig. 1:**
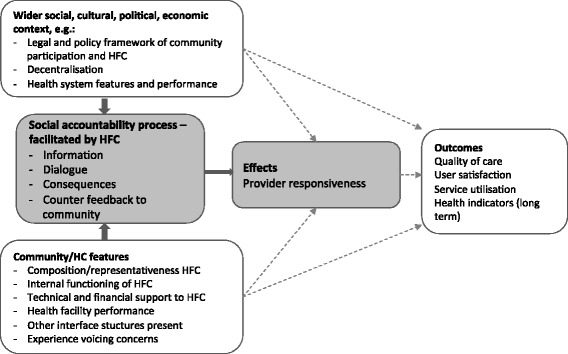
Conceptual framework

This paper explores the activities HFCs currently perform in providing a social accountability interface only (4th “outward role” in Table 2) that are summarised in four steps: information/data collection, dialogue/forum, consequences and counter-feedback to users [15]. In line with Bovens, HFCs provide a “forum” where they question health providers’ behaviour and actions, and where health providers provide explanations or justifications; when such explanation or justification fails, consequences (sanctions or rewards) can follow [15]. We added the fourth step “counter feedback to users and citizens” as HFCs are representative of the communities in which they operate and have, themselves too, the obligation to report on their activities and results to the larger community. Apart from assessing the functioning of the accountability cycle in the study countries, we also aimed to explore the effect on health providers’ responsiveness to community issues and demands and the factors that shape HFCs as social accountability interfaces. Figure 1 summarises the conceptual framework for the study. It combines concepts from the work by McCoy et al. and Molyneux et al. on HFCs, and from Bovens’ accountability theory [4, 9, 15]. This framework supported the data collection and analysis.

The four steps of the accountability cycle are expected to affect health provider responsiveness, an intermediary effect of social accountability initiatives [16]. Responsiveness broadly refers to the extent to which a health provider or health policymaker acts upon needs and demands expressed by users, the community or HFCs [16]. The ability of HFCs to influence provider responsiveness and service provision depends on several interacting factors related to the wider social, cultural, political and health system context and the local context regarding community and HFCs features [4, 9, 10, 15, 17]. In this study we were interested in one particular community feature: the presence of other interface structures such as community health workers or local councils. We assumed that other community groups, also those beyond the direct environment of health facilities, demand accountability and advocate for improvements in health services and hence influence the scope and reach of HFCs [18].

Based on the conceptual framework, the three main research questions of the study are: how is the accountability role of HFCs described in policies? How do HFCs currently facilitate social accountability? What factors shape the role of HFCs as facilitators of social accountability?

## Methods

This article is based on findings from three country studies that were conducted in 2013 and 2014 in Benin, Guinea and the Democratic Republic of Congo (DRC) [19–21]. The studies were initiated in the context of the French Muskoka Initiative with UNICEF WCARO that funds operational research in Francophone countries in West and Central Africa to support the emergence of innovative approaches to health systems development. The study adopted a qualitative case-study methodology to understand social accountability practices in selected primary health care settings within their real-life context [22].

### Selection of study sites

The countries were purposively selected; criteria included francophone countries, government commitment to the Bamako Initiative, community participation and HFC development and long-term experience with HFC programmes. In each country, districts (*department* in Benin, *zones de santé* in DRC, *prefectures* in Guinea) were purposively sampled taking into account the presence of community participation support programmes. In Guinea, two prefectures were selected relatively close to the capital Conakry because of the Ebola epidemic that had not yet reached the capital at the time of the fieldwork.

Within the districts, four health centres were selected based on their health performance. Based on suggestions by McCoy et al., we assumed that well-performing facilities could be associated with well-performing HFCs [9]. We used health performance indicators as selection criteria (external consultation, assisted deliveries and antenatal care coverage). Not all provinces disposed of performance data, therefore in some cases; the researchers depended on the knowledge of health authorities. The facility closest to the national capital was at 60 km (Guinea) and the most remote facility was at 220 km. from Kinshasa (DRC). Initially, each country team had selected four study HFCs; in Guinea, the dataset of one HFC got lost, totalling the total number of study sites at 11.

### Data collection and analysis

In each country, a research team was set up consisting of a senior researcher, a researcher and research assistants. In Benin, the Belgian Technical Cooperation (BTC) participated in the design of the study. The overall study was coordinated by the Royal Tropical Institute (KIT). Senior researchers participated in the protocol and tool development and organised training for the research teams in their respective countries. Data collection took place between January and April 2014. Each country team had a backstopping advisor from KIT, who supported data collection, analysis and feedback on interim reports.

An initial review of the health, decentralisation and community participation policies helped to understand the background of HFCs, the evolution of the Bamako Initiative and the formal arrangements about the role of HFCs in social accountability. Secondly, to investigate the practices of HFCs in facilitating social accountability, we used a mix of methods including semi-structured interviews, focus group discussions and document reviews, each with specific tools. Each of the tools addressed three main topics: (1) characteristics of the HFC and the health facility (local context), (2) examples of social accountability practices, and (3) participants’ perceptions of the effectiveness of social accountability relations and practices. The triangulation of different sources of evidence to answer the same questions was expected to strengthen the validity of the findings [22].

For the interviews, we targeted participants at each health facility site representing health providers, local authorities, HFC members, community based associations and users (male and female) in order to collect a diversity of views on, and experiences with, the HFCs. A total of 95 individual interviews and 22 focus group discussions were held with different actors (see Table 3). The selection and recruitment process differed between the countries, depending on prevalent local procedures. In DRC, the health zone director communicated to the HFC chairman who selected participants based on criteria and numbers set by the researchers. In Guinea, a site visit prior to data collection supported the identification of participants and the issuance of formal invitations through the local authorities. In Benin, the identification and recruitment of participants was done by the HFC chairmen, based on the selection criteria provided by the researchers. Some documents were collected locally including minutes of HFC meetings and health facility reports. Not all HFCs disposed of written material.

**Table 3:** Data collection method per country

Country and no. of HFCs	Individual interviews	Focus group discussions: 6–12 participants each
Benin 4 HFCs	33 Health provider (*n* = 12) Local authorities (*n* = 6) HFC members (*n* = 12) Other interface structure (*n* = 3)	6 Male citizens (*n* = 3) Female citizens (*n* = 3)
Guinea 3 HFCs	28 Health provider (*n* = 9) Local authorities (*n* = 7) HFC members (*n* = 9) Other interface structure (*n* = 3)	6 Male citizens (*n* = 3) Female citizens (*n* = 3)
DRC 4 HFCs	34 Health provider (*n* = 11) Local authorities (*n* = 9) HFC members (*n* = 14)	10 Male citizens (*n* = 4) Female citizens (*n* = 4) HFC members mixed (*n* = 2)

All individual interviews were audio-recorded and transcribed in Word. The transcripts of interviews held in local languages, were translated into French. Coding was done based on a coding guide developed jointly by the three research teams based on the conceptual framework and research questions. The coding was done through software Atlas-ti in Benin and the DRC and in Guinea in Microsoft Excel. The country research teams carried out the analysis and triangulation of country-level data. Each HFC was described as an individual case during a workshop in June 2014 where the researchers also jointly identified the first common patterns in the findings. This paper is based on a qualitative cross-case synthesis [23] that considered the findings of the country studies collectively and aimed to identify, from 11 case studies, common patterns and trends in the way HFCs perform a social accountability interface role. Authors synthesised this data from all three countries on the basis of the country research reports, the initial joint synthesis and their access to the primary data. The activities that are performed by HFCs as accountability interfaces were selected for inclusion in this paper based on a review of their clarity in describing the activity and its effect and on the basis of data (respondent) and data source triangulation. At least two groups out of four participant groups needed to have contributed to the example or the example needed to be confirmed by documentary evidence. Data about contextual factors were derived from the country reports but also by returning to the original data. To identify patterns across the countries and to include the relevant data in this analysis, a regular return to data was needed as well as some re-coding, in particular of contextual factors. Hence, the analysis was an iterative process of explanation building as proposed by Yin [22]. Investigator triangulation and team discussions contributed to the interpretation of themes and the identification of patterns across the cases, progressively leading to a better understanding and confidence in the synthesis findings [24]. Information on policy and legal country contexts was derived from previous outputs of the country studies and external literature. Regular validation of findings between the main author and the country research teams was done to ensure the quality of the results.

## Results 

The first section describes the political and legal context and the evolution of health facility committees in the three countries. The second section discusses the interface role that HFCs currently play, and the last section discusses how contextual factors shape the accountability role of HFCs.

### Evolution of HFCs in policies in Benin, Guinea and DRC

In Benin, Guinea and DRC, different types of health committees have been set up from 1979, first as local initiatives and later scaled up to other parts of the country. In all three settings, the health centre level is the lowest level that has an HFC. District or regional hospitals usually have advisory boards or boards of directors but they do not have as main task to enhance community involvement. Table 4 presents the key features of current HFCs attached to primary health care centres.

**Table 4:** Features of health facility committees attached to primary health care centres

Feature	Benin	Guinea	DRC
Denomination	Health Facility Management Committee (COGECS)	Health Management Committee (COGES)^a^	Health Development Committee (CODESA)
Installation	1987 (first committee) 1995 (roll out in whole country) 2004/2006 updated bylaws	1990 (IB policy)	1979 first HFC, 1982 expansion 2006 (new policy)
Number of HFCs	587	410	8126 (against 8504 planned)
Catchment area of rural HFC (norm)	5000 – 15,000 inhabitants	5000 – 10,000 inhabitants	5000 – 15,000 inhabitants
Membership	9 members, elected for 3 years through general assembly; representing community, local associations, health workers and local council	9 members, elected for 2 years, representing civil society, religious leaders, women, youth. The facility manager represents the health workers.	10+ members, elected for 2 years, representing community health workers (chair), civil society and the health centre Officer in Charge (OiC). The OiC cannot be a member of the HFC executive board.

Sources: Benin [25, 45], Guinea [28, 46, 47], DRC [48, 49]

^a^Also called “Comité de Sante et d’Hygiene (CSH) but for the purpose of clarity in this paper, the term Health Management Committee is used

The table shows that the three countries have HFCs of similar compositions. They all have community as well as health worker representatives, and around nine members who are elected for two to three years. Health worker representatives cannot be elected as board members or chairpersons but can act as secretaries. HFC compositions differ in that guidelines in Benin and Guinea prescribe the need to include representatives from different social groups, while in DRC community health workers form a large proportion of the membership. In the three countries, there is a clear policy commitment to community participation and reference is made to HFCs in many health sector strategies as well as health system strengthening plans.

In Benin, community participation is considered a crosscutting issue and is integrated into different health policies and reform documents. Since the Bamako Initiative, several types of community structures have been installed; it is the health facility management committee (COGECS) that is assessed in this study, further referred to as HFC. After the countrywide installation of HFCs from 1995, the Ministry of Health further defined the mandate and powers of the HFCs in 2004 and 2006. Bylaws stipulate that HFCs are involved in the monitoring of the budget formulation and execution, the management of user fees, and the establishment of drug inventories and orders. The texts specify that HFCs are to promote financial transparency of pricing policies and to prevent extortion of patients and illegal drug sales. They have to report health workers suspected of fund diversion or embezzlement to the authorities to enable disciplinary measures. HFCs are further supposed to contribute to conflict resolution between the community and health providers [25, 26]. The Ministry further suggests strengthening the ethical behaviour of health workers through the training of user associations [26]. For each of the different functions, a training manual exists with concrete suggestions for conflict management and financial control [27].

In Guinea, the Ministry of Health, in its National Health Development Plan (2004), envisions communities as owners of health facilities instead of clients. The Ministry envisions the development of formative supervision and participatory monitoring with communities to enhance the quality of primary health services [28]. HFCs are supposed to plan and monitor health services at health facility level. They also engage in supervision, including the checking of health workers’ presence and quality of the reception of patients. The HFC training manual emphasises that supervision is meant to solve problems rather than to control or inspect [29]. HFCs are expected to “maintain a continuous dialogue” between health providers and the community to reduce conflicts and to transmit perceptions and expectations of the community to providers. Community participation policies in the health sector are increasingly aligned with the overall decentralisation policy that established local governments from 1988. This means that HFCs are now under the authority of local councils and are accountable to local councillors and the mayor. A government-adopted training manual of 2011 reflects this set-up; it describes responsibilities of both HFCs and the health, political and administrative authorities [29]. After this study, the health system has undergone many changes as a result of the Ebola epidemic, whereby the role and mandate of HFCs are also being revisited.

Community participation in DRC has been revitalised in 2000 as a result of a health sector review that observed continued high mortality and morbidity rates. The Ministry of Health proposed to transform the ineffective or non-existent health management committees into multidisciplinary, multi-sectoral health development committees. The new HFCs are strongly associated with Community Health Workers (CHW) who play an important role in health care in the DRC in the context of a shortage of trained health professionals [30, 31]. Each village has a CHW representative in the HFC. The HFC manual lists a range of responsibilities for the HFCs including planning and monitoring of health activities in collaboration with health professionals [31]. The integration of this governance reform in other health-related policies is yet to be realised. The implementation of the new policy is uneven with some districts benefiting from NGO support and others not.

The data presented here show that the policy framework of HFCs in the three countries has been revised in the last decade, approximately 15 years after the Bamako Initiative. This revision mostly concerned a more precise definition of the mandate and responsibilities of HFCs. In many countries, including in Benin and Guinea, the Bamako Initiative was initially interpreted as a cost recovery mechanism but the revisions in Benin and Guinea re-emphasize the role of communities in controlling the management of health facilities which includes not only financial control but also the monitoring of the quality of care [6]. The revision in DRC is more driven by a renewed commitment, in particular of external donors, to strengthen the position of community health workers (CHW) in primary health care.

For the three countries, the central role assigned to HFCs in most official documents as well as training manuals is related to the role that we classify as the “inward” role to support the facility and health workers. Despite increased commitment in new bylaws and health policies to strengthen the “outward” role of facilitating social accountability, the researchers did not find specifications of the actual powers and tools for HFCs to take up this role. For example, in both Benin and Guinea, HFCs are expected to monitor health service delivery but ways to address, report or sanction poor service delivery, are not defined. When roles and powers of HFCs to hold health workers to account are only partially defined, it can be expected that many HFCs do not take up this role or develop an approach based on their own experience and practices. In the following, we will focus on these practices to facilitate social accountability according to the four steps of the accountability cycle presented in the framework.

### HFC practices in facilitating social accountability

Table 5 provides a summary of the study findings with regard to activities carried out by HFCs within each of the accountability steps. A more detailed account of these steps is provided in the paragraphs that follow.

1. Information and data collection

**Table 5:** Summary of activities HFCs perform as accountability interfaces

Accountability interface steps	Activities
Information/data collection	Direct observation and supervision in health facilities Users approaching individual HFC members
Dialogue/forum	Direct and immediate problem solving Direct - during HFC meetings
Consequences – follow-up	No follow-up or follow-up with no results Local regulation Involvement of health authorities
Counter feedback to community	No activities

HFCs collect information on the performance of the health facility in two ways: through direct observation, monitoring and supervision in health facilities and through users approaching HFC members.

The first method, direct observation and supervision, is strongly related to the traditional role that HFCs have been attributed in the Bamako initiative. Although the three countries followed different paths in implementing the Bamako Initiative, the task of controlling drug management and the financial books at health facility level is key in most of the studied HFCs. An HFC chairman in Guinea illustrates the determination of many HFCs in this domain with regard to supervision of the delivery and management of drugs:

*“We control workers’ presence in the health centre and drug prices. We have achieved results such as compliance with drug prices. The health workers were planning to buy and sell the drugs and collecting money without involving us. We demanded our participation in the ordering and reception of the drugs and the deposit of amounts received from drug sales before signing any document”* (HFC chairman Guinea, HFCG1).

Besides this core task, in many cases, HFC members are present in the health facility on a daily basis to monitor the quality of care. Through their interaction with patients, they collect and share information about the health services, but they do not document their interactions.

A second way in which HFCs obtain data on health worker performance is through users and citizens approaching individual HFC members directly on the street or in other public spaces or during health information and communication sessions. The latter strategy is particularly prevalent in DRC, where HFC members (including CHW) are conducting home visits for sensitization and public health data collection purposes. Users and companions (who escort patients) use this opportunity to share their concerns about the performance of the health facility without explicitly being invited to. In Guinea, respondents give examples of individual HFC members treating complaints on the spot on a case-by-case basis. Table 6 presents the main issues brought forward by users to HFC members across the 11 cases, in order of importance and triangulated by data from users, health workers as well as HFC members.

**Table 6:** Complaints brought forward by users to HFC members

Material and financial issues	Health worker performance
High drug prices	Availability of staff
Lack of drugs, equipment	Absence of staff resulting in non-treatment (day)
Quality of drugs (illicit or wrong drugs)	Task shifting (assistant as manager instead of doctor)
Overbilling of drugs	Absence of staff (night)
Financial accessibility in case of emergency	Unfriendly behaviour of health workers and auxiliary staff
High consultation fees	Quality of reception/welcoming of patients who arrive at the health facility
Financial harassment/informal payments including: - direct cash payments to staff where the transaction is not recorded and patients are either not given a receipt or are issued a false one - double payment (by the health worker and at the official cash payment point)	Detention/bribing of patients who cannot pay their consultation fees
Lack of blood	Inebriated health workers

In none of the study sites, HFC members pro-actively seek users’ opinions or ask people to share needs, demands, expectations or complaints about health services. Tools such as patient satisfaction forms are not in use or are not managed or accessed by HFCs.

Some HFCs combine methods such as in a case in Guinea where members are present on a daily basis to monitor the quality of care, and to discuss directly with health providers in cases of poor performance. This active supervision is more likely to happen after a user complaint is received.

2. Dialogue/forum and effect

We identified two ways HFCs use to channel concerns and complaints to health providers. One is the direct pathway whereby health providers are contacted directly, mostly individually; the second is the use of HFC meetings to discuss issues. The responsiveness of health providers to these dialogues is also assessed.

#### Individual dialogue

Transmission of community concerns directly to providers occurs face-to-face or by telephone. Participants shared a number of examples whereby HFC members confront health providers immediately after having received a complaint (Benin, Guinea). An officer in charge of a health facility and a female participant in Benin stated for example:

*“When they observe awkward behaviour, complaints from the village, they call me on the phone…and I go..*. *So this is how they behave and … how they do their control…either by phone and I will come and check”* (OIC, Benin, HFCB3).

*“There are no arguments between health workers and patients here, and if it is the case, the HFC intervenes. HFC members calm everyone, they manage the situation and peace returns”* (FGD women, Benin, HFCB3).

Financial accessibility is a major problem for many patients in the three countries, especially in systems where patients have to pay before they are treated or where informal payments are widespread. Complaints about informal consultation fees and high drug prices are very common in the three countries as observed from the FGD with users and HFC members. HFCs seem to deal with these complaints in different ways. They facilitate access by convincing health workers to provide services by guaranteeing the payment, or by negotiating a credit system as the examples illustrate:

*“On a Saturday we were working at the health centre, a mother brought a child with anaemia. She did not have much money so that the pharmacy did not want to give her a blood bag for the child. Members of the health committee, who noticed this, vouched for this woman and have promised to pay if the woman did not pay. The pharmacy gave her the blood bag and the child was transfused”* (FGD HFC members, DRC, HFCD4).

#### Dialogue in HFC meetings

Another way of addressing complaints regarding the quality of care that the HFC receives is through HFC meetings. From both interviews, FGD and minutes of HFC meetings, it was observed that HFCs regularly discuss two main issues: health worker behaviour and drug management. Behaviour concerned absenteeism, informal payments, inebriation and unfriendly treatment of patients. Service users in both DRC and Guinea mentioned they had reported absenteeism to the HFC and they were aware of the HFC having addressed the issue in their meetings. According to women in an FGD in DRC, this resulted in nurses being more quickly available in emergency cases and in Guinea the officer in charge has developed a publicly displayed table with staff working hours that is, according to both female users and the officer in charge, being respected. Some respondents in DRC explain that by openly discussing health worker behaviour during a HFC meeting, frustrations from both sides are shared and listened to and that this process helps to address poor behaviour, at least in the short term. There was one case in Benin where the good collaboration between HFC members and health workers even got its own proverb “there is no problem” (*« Toukada mou léo»)* which refers to a mutual commitment to rectify problems and not let the oil stain*.*

In Benin and Guinea, HFCs often prioritise drug pricing and related problems such as embezzlement or overcharging in their HFC meetings. Some HFCs meticulously collect health passes, drug prescriptions and patient bills and compare with similar bills and with official prices. When there is visible proof (e.g. written bills), and when verification is possible, HFCs bring a strong case to the table that health managers cannot ignore. In Benin, a facility manager explains the dilemma’s this poses to him:

*“When they [HFC] call me to come and check the bills at the dispensary…I must say that I try to save my agent…I cannot undress my agent in front of them like that because it’s a secret between us. Or they [HFC] come directly to surprise us at a meeting with certain bills, I then give the floor and try to calm the HFC while scolding the agent who is at fault…we arrange it together. But last time it almost escalated, and if it were not for our vigilance, they would have locked someone. We were angry with them, we must defend the culprit”* (OIC, Benin, HFCB3).

While the systematic collection of evidence to detect fraud is practised by some HFCs, the collection and transmission of evidence on non-financial matters (e.g. health worker absenteeism or behaviour) is less systematic in the study sites and seems to be acted upon on a case-by-case basis.

3. Consequences and follow-up

As suggested in the previous section, sometimes dialogue and on-site mediation between HFC members and health providers ease frustration. Explanations for behaviour, for example by the facility in charge, may reduce tensions and solve a case. In a number of instances, however, HFCs pursue their quest for change by introducing local enforcement methods or by involving district health authorities.

#### Local regulation

Strong levels of negotiation and local regulation and enforcement were observed in two sites. In one case in Benin, a HFC introduced regulations and sanctions to enforce health providers’ financial accountability. These included the formal interdiction to sell parallel drugs using the health facility prescription orders or to sell drugs on credit without approval by the HFC. The HFC further decided that health workers who had issued false bills had to repay the debts to the health facility, which some of them have started doing. Finally, the HFC issued a warning that health workers who would fail to apply these rules would be transferred elsewhere. One HFC in DRC had appointed one member as complaint manager who had the task to follow-up the decisions made and actions identified during HFC meetings and to keep them on the agenda when the facility in charge failed to implement them. Although health providers in most sites appreciate the contributions of HFCs in co-management, they were less receptive of HFCs engaging in monitoring consultation payments and fraud detection. In Benin, an HFC member believed that providers “*can threaten to kill HFC members over too much scrutiny*”.

#### Involvement of district authorities

Community demands or complaints often move up the hierarchy after having been discussed in the HFC meeting. In Guinea this process seems most formalised whereby the transmission of complaints follows a route from the communities to health posts to health facility managers and up to the district authorities, written down in the monthly monitoring reports:

*“During some meetings, HFC members get to discuss populations’ complaints. An example is a situation of a health post, which was debated even on the management board. The officer in charge did not get along very well with people because he came to work inebriated. This complaint was sent to the HFC and sent up to the communal council, the health facility and the District Health Team. The worker was eventually replaced”* (OIC, Guinea, HFCG2).

Similar examples were reported In DRC, where poor health worker attitude was discussed by HFCs and reported to village chiefs or health authorities, first orally and then written:

*“There was a health worker, who had a habit of overcharging services and who did not treat patients well. Several community members reported these complaints to members of the health committee. The committee spoke to the provider, who, instead of changing, continued to act in the same way. Finally, after several complaints, the health committee sent reports to Health Zone Management Office who sacked the health worker in question?”* (FGC HFC, DRC, HFCD1).

*“Previously, there was a provider here at the health centre. He did not touch the patients, he used a pen to feel and inspect a patient. He did not use his hands like other providers. The people saw it and forwarded a complaint to the central office through the health committee and promised to report this attitude to higher instances or make a court case. This provider was transferred elsewhere”.* (FGD men, DRC, HFCD1).

In similar cases, however, the HFC has not been able to trigger sanctions:

*“A recent complaint we received was about a vaccination officer who was often inebriated at work… we have repeatedly informed health officials verbally before writing a letter, but so far they have not found a solution…Because it is the State that affects its agents and we often mention in our reports, but in vain. The state must resolve the complaints because it is the state that affects workers in communities”.* (HFC Chairman, Guinea, HFCG1)

In the cases mentioned, the main result is a transfer of the health worker to another health facility or the dismissal of a health worker. One HFC in Benin took a different approach to deal with health workers overcharging patients. The HFC required health workers to pledge adherence to the jointly established price list for drugs and to accept the working conditions at the health facility. The pledging was done in front of the district health authority as to enforce the use of this agreement.

4. Counter feedback to community

The last step in the accountability cycle, counter feedback to the community, does not seem to be practised. When community members report complaints to members of the HFC, most of them indicate they do not know whether their concerns reach the providers. Hence, they do not know whether the HFC members are reactive themselves or whether it is just a lack of counter feedback to the community, even if the concerns have been treated. Some HFC members confirm that they do not report back to the community; instead, they expect users to see the achievements of the HFC when visiting the health facility.

### Factors shaping the role of HFCs in facilitating social accountability

With regard to the wider context, the countries share similar policy and legal contexts whereby there is a political commitment to community participation and social accountability by HFCs but limited support and legitimisation through legal tools or practical guidelines. This may explain the observation that none of the HFCs studied applies the accountability cycle in a systematic (regular) or complete (all four steps) way. HFC members and others may not be aware of their role or not have the appropriate instruments and power to perform the role. On the other hand, the findings suggest that despite a disabling legal and political context, the HFCs in the study areas seem to use the limited space or develop the necessary approaches locally to facilitate social accountability. In the following section, we explore the factors that shape the potential of HFCs to provide an accountability interface. It is based on a comparative analysis of the 11 HFC cases presented in the country studies. It addressed three factors: the election and representation of HFCs, remuneration of HFC members and the presence of other interface structures.

#### Mode of elections, composition, representation, leadership

In all cases, HFCs were installed through an official event, but in none of the cases, HFCs were elected as anticipated in the regulations. Even in cases where elections had taken place in a transparent manner, resulting in an initial representation of different groups in the community, the composition changed soon after installation. Members who were not active or members who are believed to be incompetent or too old were soon replaced. In one HFC in Benin, for example, the Chairman replaced a treasurer deemed “too uneducated”. In two other HFCs, a locally elected councillor took the place reserved for an NGO representative. Sometimes new members are added without a consultation or vote within the HFCs. As a consequence, sometime after the elections, the composition has changed significantly, and in practice, only the executive board (chairman, treasurer) is active. Hence, the mode of election does not seem to be the problem but the recomposition of HFCs that occurred in all cases post-election. In some cases, it led to internal oppositions and unsolvable tensions between members, for example in struggles over the treasurer function. These tensions were also observable during group interviews in the study sites in Benin and Guinea. In others, it led to individualism of members (Benin) and apathy. In two cases, HFC leadership seemed to mitigate these problems; a capable and active chairman or board who insist on actively engaging with the community and providers.

#### Remuneration

In all three countries, participants mentioned the lack of remuneration for HFC members as an obstacle to the proper functioning of HFCs. In the case of Guinea, however, where HFC members are not paid and where external technical and financial assistance was largely absent, HFCs had similar levels of activity like the ones in Benin that had been receiving support for some years. Participants explained this intrinsic motivation of HFC members as a result of long periods of conflict and health facility destruction in that particular area. They argued that citizens had developed a greater sense of responsibility to manage their affairs. In one site in DRC, HFC members developed their internal operation manual in the absence of government-provided guidelines and, instead of receiving institutionalised support, they received occasional voluntary contributions from communities or individual deputies.

#### Community features: existence of multiple interfaces

As suggested in the conceptual framework, the presence of other interface structures and in particular their credibility in the eyes of the users and citizens can also influence the use and effectiveness of HFCs as accountability channels. The study aimed to explore this by asking respondents about the presence and performance of other participatory structures.

Although HFCs in the three countries are mentioned as the dominant formal structure providing “the bridge” between users and providers, other interface structures or persons play a similar role.

In Benin, respondents mentioned a large variety of actors such as mutual health insurance associations, women’s and youth associations, NGO’s, and community health workers, but also village leaders and local administrators (“chef d’arrondissement”). In a health facility that had a passive HFC, it was the village council that received and managed complaints. Participants perceived community-based health insurance structures (“mutuelles de santé”), introduced in the 1990’s, as potential strong negotiators but they cover only 5% of the population and hence might not represent the larger community. Participants also mentioned district level platforms in the context of performance-based financing as potential accountability facilitators.

In Guinea, respondents cited the role of elected councillors in local government as well as prefects (government representatives). In Guinea, the HFCs operate synergistically with other local associations, local government and authorities. The democratic decentralisation process in Guinea, introduced since 1990, seems to have led to institutionalised forms of local decision making, as expressed in many examples given by respondents. They are part of larger government efforts to decentralise governance to the local level and act jointly with local governments, education committees, etc. to strengthen the democratic base. Respondents suggested that the strong formal link between HFCs and local government empowered the HFCs and enforced local decision-making.

In DRC, respondents mentioned community health workers and village chiefs and in some areas religious leaders. There seems to be an overlap between HFCs and CHW; CHWs perform health interventions, and they constitute channels through which users share their problems and complaints, as they are members of HFCs. CHWs are generally better known as health workers than as HFCs members. Some respondents appreciated the direct effect that village chiefs could have on providers’ behaviour because of their traditional and perennial authority, but they were less positive about the accountability of village chiefs to the community. Participants mentioned religious leaders as emerging actors in health, primarily used to transfer health messages from providers to the community because of their influence on the community. An HFC member expressed that, regardless of who plays an interface role, there is a need to have a structure like the HFC to bridge the interests of “foreign” health workers and local communities:

*“If there are no health committees, providers can deviate, misbehave or destroy infrastructure because providers are foreigners, people from elsewhere who are assigned to the health centre and one day they will leave but the centre will remain. The committee consists of villagers who do not want the centre to be destroyed”* (FGD, HFC member, HFCD1).

Although users recognise the need to have an interface structure, they explain that such an interface is only useful to them if they respond to a certain number of criteria. When respondents were asked to identify key characteristics of a “good” interface they mentioned the ability to show leadership and authority vis-a-vis providers; transparency in management and decision making; and the ability to accompany users in the facility. Women in Benin, in particular, emphasised this latter aspect by stating that HFCs main role should be to *“alleviate the pain and anger patients live with as a result of unfriendly treatment and poor services at health facilities”*. If they fail to do so, they argued, users are even more inclined to avoid the health facility.

## Discussion

Community participation for the purpose of accountability and improved health facility governance has not traditionally been a major component of health programs and evaluation studies. This study aimed to explore the role of Health Facility Committees (HFC) in providing a forum for social accountability in Benin, Guinea and DRC. The findings show that HFCs address access and quality failures in health service delivery through the reception of users’ complaints and regular interaction with health facility managers and workers. The way in which HFCs collect, translate and present concerns and complaints to service providers is sometimes ad-hoc and informal but in some cases more or less institutionalised, for example through HFC meetings. In the absence of (known) formal guidelines or procedures, some HFCs have instituted forms of local regulation to address misbehaviour or fraud by health workers. Through such local dialogues and measures, some improvements are reported such as improved health worker presence, the availability of night shifts, and the display of drug prices. The HFCs in the three countries do not have the power to impose formal sanctions or rewards to health providers in cases of poor responsiveness. Some HFCs, therefore, appeal to the district health authorities enabling the activation of administrative accountability measures of the health system. HFCs then induced the transfer of poorly performing health workers. Although a transfer may not be considered a solution to the problem, it is a formal sanction in many health systems.

From Bovens’ perspective, we can conclude that most HFCs in our study offer a social accountability forum to assess, question and judge health worker actions and behaviour and to enforce change through linkages to authorities [15]. On the other hand, we saw that the first (data collection) and the last step (counter feedback to service users) of the accountability cycle are less practised and institutionalised. This seems a missed opportunity as service users stress the need to have a structure that represents them at the health centre that acts as a “bridge”, mediator and advocate. Moreover, we saw that reporting to health authorities does not always lead to action; some cases of serious or repeated misbehaviour remain unaddressed by health authorities. Hence, there exists an accountability relation between HFCs and health providers, but its’ functioning is variable and, although effective for some cases of poor performance, not always coherent, authoritative and inclusive [15].

The observation that many social accountability actions are informal and personal in nature is consistent with findings of other studies on the topic. Molyneux et al., for example, suggest that the use of personal relationships and social networks in social accountability is more common than the use of formally instituted mechanisms [4]. Also, the confused election and installation processes of HFCs whereby positions are recomposed is a phenomenon that occurs elsewhere and regularly in local management committees in West-Africa, not only in the health sector [32]. We saw that internal reshuffling of positions might lead to individualistic rather than collective or institutionalised social accountability processes. Many issues are dealt with by an individual HFC member, and even when handled collectively, a small group of HFC members may dominate the HFC agenda. Biased representativeness, combined with limited community consultation (step 1) may translate in HFCs receiving only a fraction of community concerns about the health centre. A similar point is made by Knippenberg et al. who found that in Benin, Guinea and Mali, the voice of marginalised groups is excluded in primary health care management because of elitism in HFCs [33].

The absence of a systemic and collectively agreed feedback procedure leaves additional opportunities for concerns and issues to be overlooked. Even when HFCs actively collect complaints, there are risks that the complaints get lost because of poor documentation [34, 35]. Also, a lack of arbitration and transparency in decision-making can lead to biased or unfair outcomes, in particular when health providers are themselves involved in the issue, when they dominate HFC decision making or when politicians or ‘patrons’ interfere with the judgment or sanctioning of health providers’ performance [36, 37]. Some of these risks of biased processes and outcomes of social accountability may also apply to the HFCs in our study. We, however, recognise the tension Loewenson et al. describe between influential individuals having the leverage to engage with health providers and improve the quality of care and the simultaneous absence of the voice of more marginalised groups [38]. HFCs in this study operate within existing social, cultural and religious structures in their communities. Members often include school directors, teachers, village chiefs, religious leaders and in one case an ex-military officer, who each can draw on a form of authority and legitimacy in their interactions with health providers, even where health providers are HFC members. For example, the two HFCs who instituted forms of local regulation to call health workers to account were headed by school directors. Although power asymmetries may remain between communities or patients and health providers, they may be more balanced and less pronounced between “elite” community members and health providers. Furthermore, HFCs that have an “elite” composition may form alliances with health providers to lobby the health system and local government authorities. In their study on HFCs in Nigeria, Abimbola et al. argue that members with a high social or economic status are particularly important in contexts where HFCs receive little government or external support; they can bear the costs of participation and facilitate all the functions HFCs are expected to play [39]. The question remains how linkages and accountability relations between HFC representatives and communities, in particular, marginalised groups and women, can be ensured. The tension between representation and influence is likely to be present in other social structures such as women’s groups, local governments or health insurance associations and is not limited to HFCs; it will remain an important point in future debates and research on the nature of collective action and power relations in social accountability.

### Practical implications

The conceptual framework of this study provides a useful lens to identify barriers and opportunities for strengthening social accountability through HFCs. Our findings affirm the importance of HFCs for health service and system responsiveness and, therefore, support recent calls on governments to acknowledge HFCs in their policies and on funders and global policymakers to support HFCs’ role in the governance of health systems [8]. It is clear that, to develop more coherent, authoritative and inclusive social accountability processes and achieve more equitable outcomes, actions at multiple levels and with multiple actors are required.

HFCs need to be empowered through a more explicit mandate in the field of planning, monitoring and supervision of (clearly defined quality issues within) primary health service delivery. They need instruments to propose agreements, allow more systematic data collection and documentation and skills to engage in dialogue and feedback with health providers and other stakeholders. The consequences of a lack of responsiveness of health providers to HFC feedback need to be clearly defined. In a pilot project in Mali, for example, HFCs are empowered through a results-based contracting approach with health providers. Rather than relying on higher-level health authorities for follow-up and enforcement, HFCs have the possibility to attach predetermined rewards and sanctions to health provider performance [40]. Such contracting approach may increase the formalisation of roles and transparency of social accountability processes that is now often absent. Pilot initiatives with Community Scorecards involving HFCs in DRC have contributed to more inclusive interactions between communities and health providers [41]. Their validation, upscaling and embedding in the wider health system, however, requires more complex reforms.

Social accountability relations are part of a larger governance landscape. HFCs as accountability forums will not be successful if they cannot leverage formal sources of power such as those within the administrative accountability system. This means that district health management teams need the institutional capacity, power and incentives to supervise health workers and follow up on community concerns and complaints. Decentralisation reforms and devolved budgets enable such capacity [41]. Also, formulations or revisions of community participation guidelines need to define HFCs position and complementarity vis-à-vis other interface structures, such as health insurance platforms in Benin, [42], local governments in Guinea and CHWs in DRC. In DRC, CHWs are elected within their communities; as HFC members, they constitute an important channel for service users’ voice. Hence, through the strengthening of HFCs, the organisation of CHWs and community voice can be strengthened and vice-versa [43]. This may not apply to contexts where CHWs are employed by the Ministry of Health (where they might be perceived more as health workers than as community representatives) or where they are not members of HFCs (having limited influence in formal community structures). Hence, HFCs should be conceptualised as one accountability forum amidst other structures that can perform similar or complementary roles, each according to their competencies, position (facility-based or external) and power.

Furthermore, our findings suggest that HFCs can generate responsiveness and improved community-health centre linkages at the local level. But, as suggested elsewhere, is it not likely that HFCs engagement in local social accountability leads to the kinds of increases in human and financial resources and drugs needed for good quality of care [44]. Therefore, social accountability initiatives need to distinguish between different levels of responsibility and take into account providers’ capacities and resources to respond to concerns and claims raised by HFCs. Initiatives also need to consider how accountability practices and procedures can be respectful of health providers’ rights to transparent and fair feedback processes and consequences. Finally, a challenge in practice will be how to sustain social accountability practices through HFCs in the face of other pressing health and health system challenges. The Ebola epidemic in Guinea, for example, re-emphasizes the role of HFCs: that of health messengers and surveillance assistants. In DRC, policy discussions revolve around the need to develop more intersectoral committees to address the broader determinants of health. In the context of changing needs for community participation, HFCs will be asked or required to shift priorities and navigate their multiple roles. In such contexts, it will be important to reflect on ways in which feedback loops and accountability forums can be maintained at the local level to ensure that poor health worker practices are addressed and to promote people-centred health services.

### Strengths and limitations

This study contributes to a further understanding of the potential of HFCs in strengthening health provider accountability and responsiveness. Although the findings present just a fraction of a whole range of practices and factors characterising the interaction between service users, HFCs and health providers in the study sites, they provide a synopsis of current practices and challenges of social accountability across three countries. In research synthesis, decisions are made on the most relevant aspects to cover. The team took such decisions iteratively with key moments of consultation between the researchers and the authors. The final set of themes covered in the paper, however, is more a subjective choice of the authors and therefore they may have disregarded important themes to the three country contexts. Furthermore, although coordination and quality assurance measures were in place, the country research teams worked in different contexts and with varying qualitative research experience that might have influenced the quality of the interviews, and the type of responses researchers got. Finally, because the initial research questions focused on the potential accountability role of HFCs and in interviews we inquired about examples of ‘good’ practice, there may be a bias in the results; the data provided more positive examples than expressions of dissatisfaction with HFC performance.

## Conclusions

This qualitative study explores the ways in which HFCs in West and Central Africa facilitate social accountability in primary health care. The findings confirm that many barriers to the quality of daily service provision can be addressed at the frontline of service provision even if accountability relations are poorly defined in policies or operationalised in guidelines. The study concludes that policymakers and funders should recognise and further support the role of HFCs in promoting responsive health services. For HFCs to facilitate social accountability in an inclusive and sustainable way at the operational level, their mandate and powers in service monitoring need to be made more explicit, and they need instruments to facilitate a full accountability cycle, in particular regarding community consultation and feedback.

## Abbreviations

BTC: Belgian Technical Cooperation
CHW: Community health worker
COGECS: Comité de Gestion du Centre de Santé (Bénin)
DRC: Democratic Republic of Congo
FGD: Focus group discussion
HFC: Health facility committee
KIT: Royal Tropical Institute
NGO: Non-governmental organisation
